# Stereospecific on‐Surface Cyclodehydrogenation of Bishelicenes: Preservation of Handedness from Helical to Planar Chirality

**DOI:** 10.1002/chem.202102069

**Published:** 2021-08-26

**Authors:** Bahaaeddin Irziqat, Aleksandra Cebrat, Miloš Baljozović, Kévin Martin, Manfred Parschau, Narcis Avarvari, Karl‐Heinz Ernst

**Affiliations:** ^1^ Molecular Surface Science and Coating Technology Laboratory Empa Swiss Federal Laboratories for Materials Science and Technology Überlandstrasse 129 8600 Dübendorf Switzerland; ^2^ MOLTECH-Anjou UMR 6200, CNRS, UNIV Angers 2 bd Lavoisier 49045 Angers Cedex France; ^3^ Nanosurf Laboratory Institute of Physics The Czech Academy of Sciences Cukrovarnická 10 162 00 Prague Czech Republic; ^4^ Department of Chemistry University of Zurich Winterthurerstrasse 190 8057 Zürich Switzerland

**Keywords:** chirality, helicenes, polyaromatic hydrocarbons, scanning tunneling microscopy, surface chemistry

## Abstract

**Flattening helices while keeping the handedness**: On‐surface cyclodehydrogenation of bishelicene enantiomers leads stereospecifically to (*M*,*M*) and (*P*,*P*) chiral planar polyaromatic hydrocarbons. This is followed by their homochiral aggregation into a 2D conglomerate. Thermally induced cyclodehydrogenation proceeds stereospecifically to chiral, planar coronocoronene. Such a reaction is a special example of topochemistry in which enantiospecific conversion is supported by the alignment of the reactant by the surface.

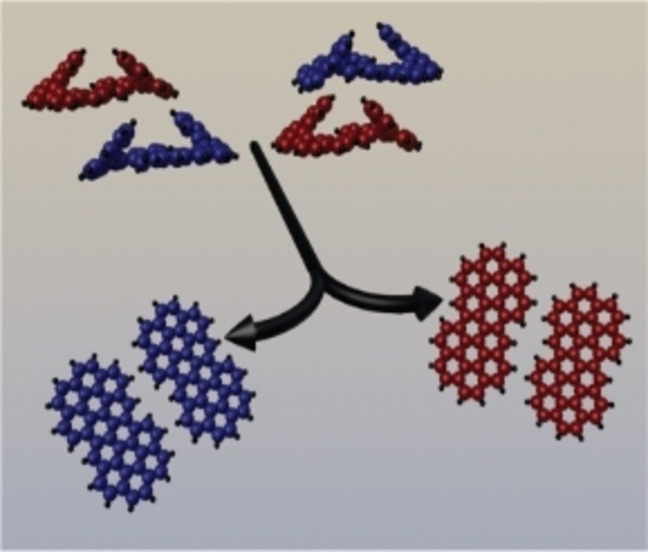

The on‐surface dehydrogenation of bispentahelicene on a gold(111) surface has been studied by means of scanning tunneling microscopy. Deposition of 2,2’‐bispentahelicene onto a gold surface under ultrahigh vacuum leads exclusively to formation of the homochiral (*M*,*M*) and (*P*,*P*) diastereomers. Thermally induced cyclodehydrogenation proceeds then enantiospecifically into planar coronocoronene, which breaks mirror symmetry due to surface confinement. Upon cooling, the coronocoronene enantiomers crystallize into a two‐dimensional conglomerate of homochiral domains.

Cyclodehydrogenation of sterically overcrowded precursor molecules into planar polycyclic aromatic hydrocarbons (PAH) is an important step to all‐carbon nanostructures, such as nanographenes.[^1^, ^2^, ^3^, ^4^] For that matter, on‐surface chemistry has become an important approach towards new functional interfaces that are hardly available by solution chemistry.^[5–7|^ For example, graphene nanoribbons, carbon nanotubes and nanographenes have been synthesized after deposition of aromatic precursors followed by thermally induced dehydrogenation and C−C coupling.[^8^, ^9^, ^10^, ^11^, ^12^]

Due to surface alignment and two‐dimensional packing of precursors, C−C coupling reactions can proceed highly stereospecifically.^[13]^ For helically shaped aromatic hydrocarbons, so‐called helicenes, diastereoselectivity has been reported for on‐surface Ullmann coupling into bishelicenes.[^14^, ^15^] Bisheptahelicene or dibenzoheptahelicene, for example, can be planarized thermally on surfaces by Diels–Alder cycloaddition followed by cyclodehydrogenation.[^17^, ^18^]

Here, it is shown that the hitherto unknown 2,2’‐bispentahelicene (C_44_H_26_, bis[5]H) undergoes cyclodehydrogenation such that the sense of helicity is converted specifically into planar chirality (Scheme 1). At first, the self‐assembly of bis[5]H on a gold(111) surface has been investigated by means of scanning tunneling microscopy (STM), supported by force field calculations and time‐of‐flight secondary ion mass spectrometry (ToF‐SIMS). After deposition of bis[5]H onto Au(111), only (*M*,*M*)*‐* and (*P*,*P*)*‐*bis[5]H enantiomers are observed on the surface. This finding is attributed to substantial steric overcrowding of a potential surface‐confined (*M*,*P*) isomer in combination with low inversion barriers of the pentahelicene subunits (Figure 1). As the distal ends of both enantiomers spiral up away from the center, C−C coupling and dehydrogenation leads to planar coronocoronene (**2**) enantiomers, which aggregate upon cooling into homochiral domains, that is, into a 2D conglomerate.

**Scheme 1:**
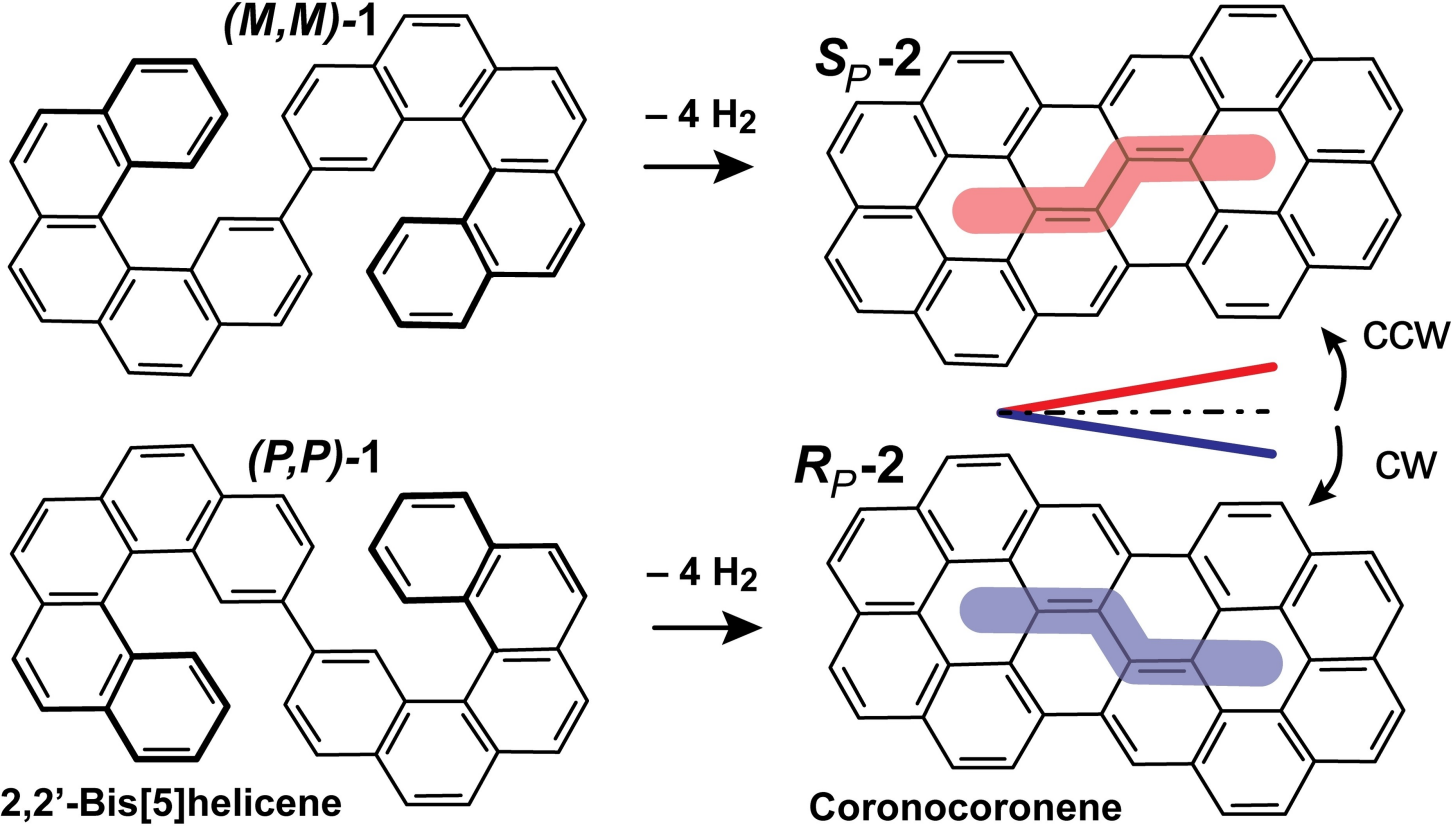
Stereospecific cyclodehydrogenation of bisheptahelicene into coronocoronene. Due to surface confinement, the product is chiral. (*M*,*M*)*‐* and (*P*,*P*)*‐*2,2’‐bis[5]H enantiomers turn into (*S_P_*)*‐* and (*R_P_*)*‐*enantiomers, respectively. (*S_P_*) stands for going intramolecularly from one coronene center to the other if one has to turn left, whereas for (*R_P_*) one has to turn right.

**Figure 1:**
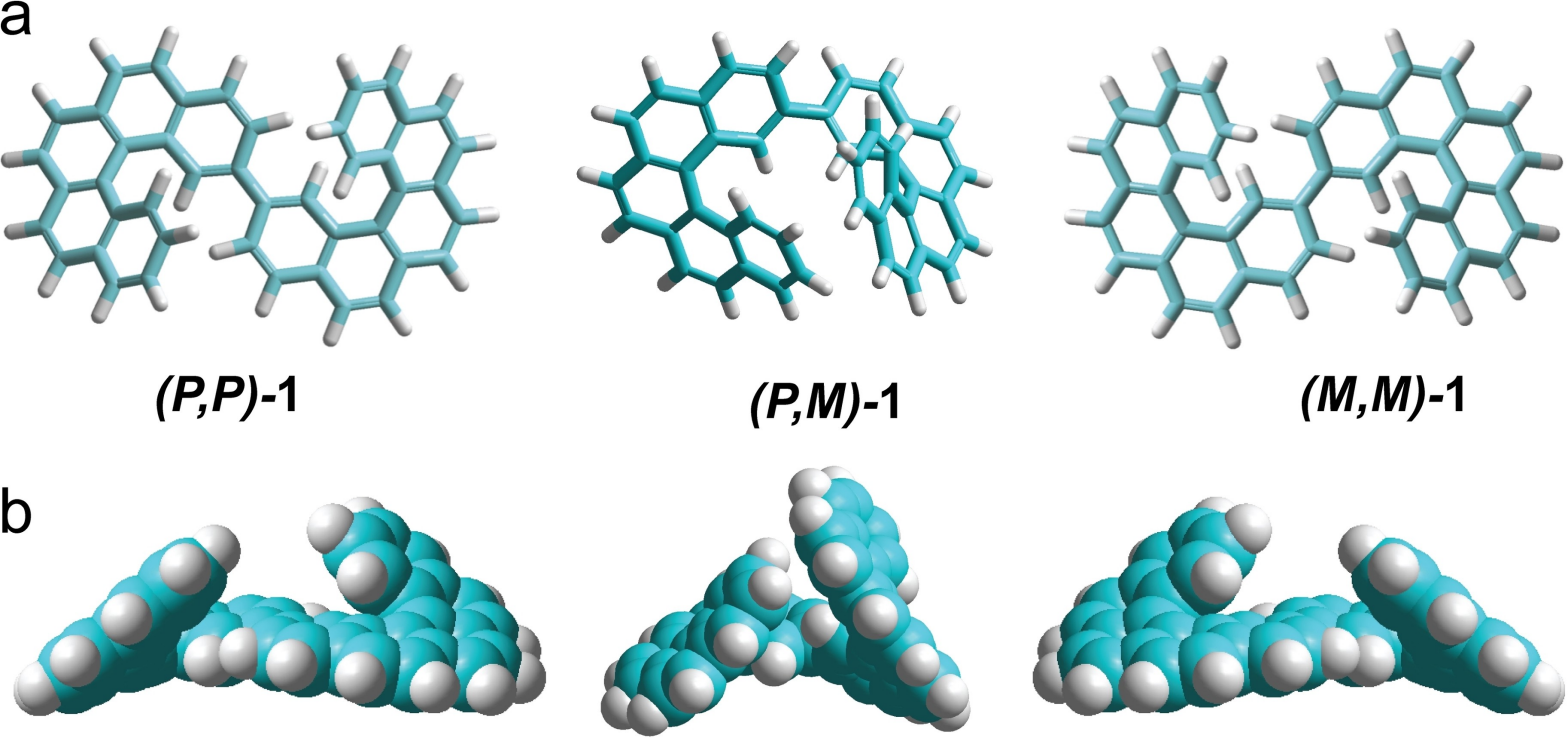
Diastereomers of bis[5]H. a) Stick models of the *P*,*P* and *M*,*M* enantiomers and the (*M*,*P*)*‐meso* form. b) Full‐space models of the diastereomers.

Details of experimental and computational methods as well as chemical synthesis of racemic bis[5]H, involving a homo‐coupling of 2‐Br‐[5]H, are presented in the Supporting Information. Briefly, the molecules have been sublimated in ultrahigh vacuum onto the gold surface held at room temperature. STM investigation has been performed after cooling the sample to 50 and to 7 K. Dehydrogenation has been induced by stepwise annealing to 400 °C.

Figure 2 shows STM images of bis[5]H on Au(111). At low coverage, the handedness of single molecules is clearly deduced by bright protrusions (Figure 2a). As these are the upper (distal) ends of the spirals, spiraling downward in a counterclockwise fashion (red circular arrow) marks (*M*) helicity and spiraling downward in a clockwise fashion (blue circular arrow) marks (*P*) helicity. Modeled electron density grayscale maps of unoccupied states of the two enantiomers fully relaxed on the surface (Figure S1 in the Supporting Information) support the assignment (Figure 2a, insets). Although all diastereomers are observed in solution, (*M*,*P*)‐ or (*P*,*M*)‐*meso* forms are not observed on the surface with STM. Landed at room temperature on the surface, the meso forms convert into the (*M*,*M*)/(*P*,*P*) enantiomers in order to maximize contact to the surface and to minimize steric overcrowding. With increasing coverage, the molecules assemble into partly ordered motifs, aligned by the Au(111) surface structure (Figure 2b, c). That is, chains form along the hcp/fcc superstructure of the reconstructed surface.^[19]^ Judging by the faint contrast near the bright protrusions, both enantiomers are found in this semi‐ordered van der Waals assembly, which is particularly evident at the end of such rows (Figure S2). At full monolayer coverage a 2D racemate crystal is formed (Figure 2d). Although the STM contrast does not unequivocally show the absolute handedness of a single molecule, the four‐lobe contrast of each entity alternates mirror‐like (Figure S2). The modeled STM contrast for a tentative structure model with both enantiomers in the unit cell (Figure S3) is in good agreement with the experiment (Figure S2). Force‐field model calculations performed for bis[5]H dimers on the surface also show a small preference for heterochiral recognition (Figure S4).

**Figure 2:**
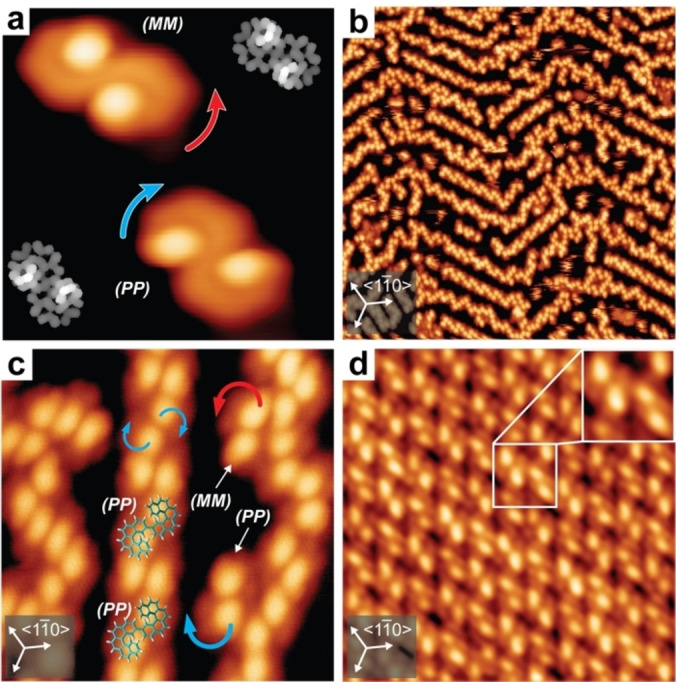
Self‐assembly of racemic bis[5]H on Au(111). a) High‐resolution STM image (4×4 nm^2^, *U*=400 mV, *I*=120 pA, *T*=7 K) showing individual (*M*,*M*)*‐* and (*P*,*P*)*‐*bis[5]H enantiomers. Curved colored arrows indicate the sense of helicity spiraling downward. The insets present grayscale unoccupied orbital density maps. b) STM image (60×60 nm^2^, *U*=−2.5 V, *I*=20 pA, *T*=50 K) at sub‐monolayer coverage showing, in part, zigzag chains oriented along the herringbone reconstruction of the Au(111) surface. c) STM image (10×10 nm^2^, *U*=−2.5 V, *I*=20 pA, *T*=50 K) of a zigzag chain segment, in part superimposed with bis[5]H molecular stick models. Circular arrows indicate the clockwise (blue) and counterclockwise (red) intermolecular helical descent deduced from STM contrast. d) STM image (15×15 nm^2^, *U*=−2.5 V, *I*=25 pA, *T*=50 K) of the monolayer saturation coverage structure. The inset (3×3 nm^2^) is a magnified cut‐out and shows different contrast for adjacent molecules. See Figure S2 for STM pattern analysis. Triple white arrows mark the orientation of the (111) surface.

Thermal treatment at 620 and 670 K leads to almost complete and fully complete planarization of bis[5]H into **2**, respectively. Dehydrogenation occurs at eight C atoms and four new C−C bonds are formed between C atoms 1 and 14, 1’ and 14’, 3 and 13’, as well as between 3’ and 13 of the bis[5]H molecule (Figure S5). Such chemistry is supported by ToF‐SIMS, showing the loss of 8 H atoms (Figure S6).

Figure 3 shows STM images of the planarization products. After treatment at 620 K, there are a few molecules that still show a bright protrusion on one side (Figure 3a). These are entities in which only one helicene subunit has been cyclodehydrogenated (Figure 3b,c). As the planar product is chiral in its adsorbate state, aspects of chirality also apply to its self‐assembly. The absolute handedness of semi‐planarized product is easily determined, and it shows strong preference to be incorporated into homochiral domains of completely planarized product with identical handedness (Figure 3d). After complete planarization, as achieved after thermal treatment at 670 K (or bis[5] deposition at that temperature) conglomerate aggregation into homochiral domains of **2** was observed. Confirmation of this scenario required submolecular resolution, which was achieved by STM studies at 7 K with a carbon monoxide modified STM tip (Figure 3e, f).

**Figure 3:**
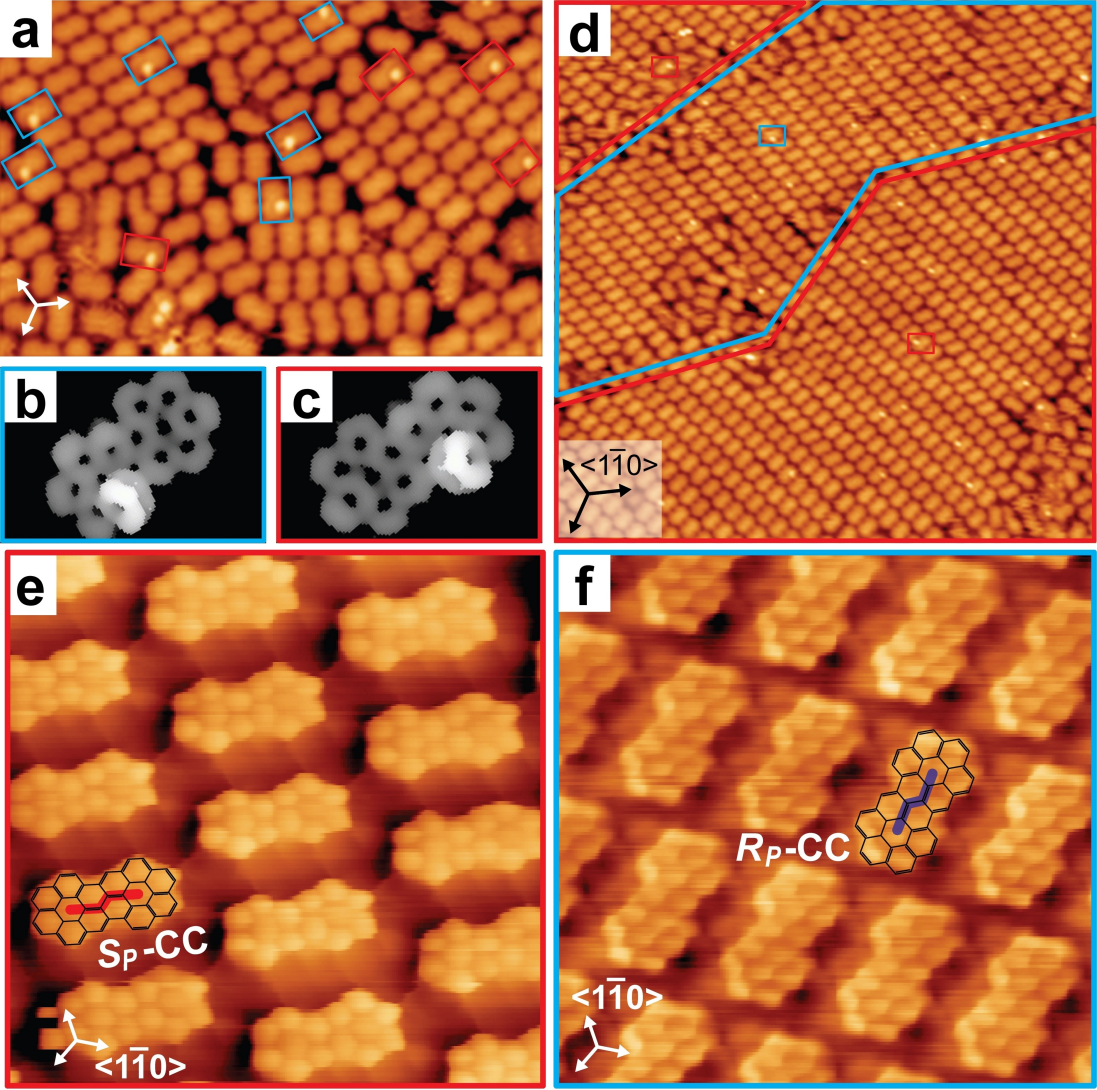
Selective formation of coronocoronene by thermally induced cyclodehydrogenation of bis‐[5]H on Au(111). a) STM image (22×15 nm^2^, *U*=1.0 V, *I*=20 pA, *T*=50 K) revealing the coexistence of mostly planar species and semi‐planarized species highlighted by red and blue rectangles after annealing to 620 K. b), c) Unoccupied states density maps of the opposite‐handed semi‐planarized products show only one bright helical subunit per molecule. d) STM image (40×40 nm^2^, *U*=1.2 V, *I*=20 pA, *T*=50 K) showing the predominant presence of self‐assembled domains of completely planar molecules. The semi‐planarized molecules suggest the presence of a 2D conglomerate. Homochiral domains are indicated by red and blue edging. e), f) High‐resolution STM images (5×5 nm^2^, *U*=70 mV, *I*=1.44 nA, *T*=7 K; and 5×5 nm^2^, *U*=50 mV, *I*=1.12 nA, *T*=7 K) acquired after annealing to 670 K. At this stage, the planarization is complete, and a conglomerate of homochiral domains of (*S_P_*)*‐* **2** (e) and (*R_P_*)*‐* **2** (f) is formed.

In contrast to bis[5]H dimers, force‐field modeling of surface‐confined dimers of **2** shows a small preference for homochiral interactions (Figure S7). However, such conglomerate aggregation is rather due to the denser packing in case of homochiral domains, thus resulting in an overall larger molecule–surface interaction. As the molecular mobility at 670 K is high, homochiral recognition occurs upon cooling at lower temperatures. Enantiomeric conversion can be safely excluded because the large contact area between molecule and surface causes strong binding. Based on previous density functional theory (DFT) studies,[^20^, ^21^] the binding energy of **2** to Au(111) is estimated to be between 4.42 and 5.63 eV. Support for the course of the reaction comes also from force‐field calculations, comparing the surface binding energies of bis[5]H, the semi‐planarized intermediate and the final product **2** (Table S1).

Each enantiomer converts exclusively to a specific product. (*M*,*M*)‐bis[5]H gives (*S_P_*)‐**2** and (*P*,*P*)‐bis[5]H leads to (*R_P_*)‐**2**; this means that on‐surface cyclodehydrogenation proceeds indeed stereospecifically. Enantiomerization of the [5]H subunits is expected to occur even on the surface at these temperatures, but a potentially forming (*M*,*P*)*‐meso* form cannot undergo complete dehydrogenation. Once the dehydrogenation starts on one side of the molecule, the chirality of the product is fixed, thus explaining the stereospecificity. Semi‐planarized product can only lead to opposite handed completely planarized product if it flips over on the surface. Again, such process is highly unlikely due to the strong interaction of planar polyaromatic hydrocarbons with the surface.

The last step of on‐surface synthesis of graphene nanoribbons (GNR) includes also cyclodehydrogenation.[^22^, ^23^, ^24^] Starting with polyanthrylene polymers, annealing to 600 K causes partial cyclodehydrogenation, and annealing at 670 K causes formation of planar ribbons. The mechanism of the cyclodehydrogenation into GNR has previously been studied by means of DFT.[^20^, ^25^] Transferring the conclusions of that study to the bis[5]H cyclodehydrogenation leads to the following reaction mechanism (Figure S8): i) Due to temperature induced vibrations of the molecular frame and van der Waals interactions, the distal end of one helix gets closer to the proximal part, enabling C−C bond formation between C atoms 1 and 14 as well between 3’ and 13. ii) The hydrogen atoms at the proximal part of the helix are then catalytically detached by the surface. iii) The upper hydrogen atoms at C13 and C14 are transferred by a 1,3 shift to rim carbon atoms, forming an intermediate CH_2_ group from which iv) the lower hydrogen atoms are also catalytically detached. Alternatively, thermally allowed 1,5‐H shifts or the involvement of gold adatoms, both not considered in the DFT evaluations, could be involved here. Because a semi‐dehydrogenated species with only one helix planarized is observed as intermediate, the first C−C bond formation must have the highest barrier with the following steps easier to complete. The fact that hydrogen at sterically overcrowded positions is most reactive is also supported by catalytic oxidation of respective polyaromatic hydrocarbons.^[25]^

In summary, adsorption of bis[5]H leads exclusively to the formation of (*M*,*M*) and (*P*,*P*) enantiomers on Au(111), whereas a (*M*,*P*)*‐meso* form is not observed on the surface. Aggregation into a 2D racemate crystal occurs at full monolayer coverage. Thermally induced cyclodehydrogenation proceeds stereospecifically to planar coronocoronene, which is chiral due to surface confinement. The product then assembles into a conglomerate of enantiopure domains. Such a reaction is a special example of topochemistry in which the alignment of reactant by the surface supports enantiospecific conversion.

## Conflict of interest

The authors declare no conflict of interest.

## Supporting information

As a service to our authors and readers, this journal provides supporting information supplied by the authors. Such materials are peer reviewed and may be re‐organized for online delivery, but are not copy‐edited or typeset. Technical support issues arising from supporting information (other than missing files) should be addressed to the authors.

Supporting Information

## References

[1] L. Zhi , K. Müllen , J. Mater. Chem. 2008, 18, 1472–1484.

[2] K. Müllen , J. P. Rabe , Acc. Chem. Res. 2008, 41, 511–520. 18410086 10.1021/ar7001446

[3] A. Narita , X.-Y. Wang , X. Feng , K. Müllen , Chem. Soc. Rev. 2015, 44, 6616–6643. 26186682 10.1039/c5cs00183h

[4] Y. Koga , T. Kaneda , Y. Saito , K. Murakami , K. Itami , Science 2018, 359, 435–439. 29371465 10.1126/science.aap9801

[5] Q. Fan , J. M. Gottfried , J. Zhu , Acc. Chem. Res. 2015, 48, 2484–2494. 26194462 10.1021/acs.accounts.5b00168

[6] L. Dong , P. N. Liu , N. Lin , Acc. Chem. Res. 2015, 48, 2765–2774. 26317241 10.1021/acs.accounts.5b00160

[7] F. Klappenberger , Y.-Q. Zhang , J Björk , S. Klyatskaya , M. Ruben , J. V. Barth , Acc. Chem. Res. 2015, 48, 2140–2150. 26156663 10.1021/acs.accounts.5b00174

[8] J. Cai , P. Ruffieux , R. Jaafar , M. Bieri , T. Braun , S. Blankenburg , M. Muoth , A. P. Seitsonen , M. Saleh , X. Feng , K. Müllen , R. Fasel , Nature 2010, 466, 470–473. 20651687 10.1038/nature09211

[9] J. R. Sanchez-Valencia , T. Dienel , O. Gröning , I. Shorubalko , A. Mueller , M. Jansen , K. Amsharov , P. Ruffieux , R. Fasel , Nature 2015, 512, 61–64. 10.1038/nature13607 25100481

[10] G. Beernink , F. Dötz , A. Birkner , K. Müllen , C. Wöll , Angew. Chem. Int. Ed. 1999, 38, 3748–3752. 10649346

[11] G. Beernink , M. Gunia , F. Dötz , H. Ostrom , K. Weiss , K. Müllen , C. Wöll , ChemPhysChem 2001, 2, 317–320. 23696505 10.1002/1439-7641(20010518)2:5<317::AID-CPHC317>3.0.CO;2-L

[12] R. Zuzak , J. Castro-Esteban , P. Brandimarte , M. Engelund , A. Cobas , P. Piątkowski , M. Kolmer , D. Pérez , E. Guitián , M. Szymonski , D. Sánchez-Portal , S. Godlewski , D. Peña , Chem. Commun. 2018, 54, 10256–10259. 10.1039/c8cc05353g 30141797

[13] H. Chen , L. Tao , D. Wang , Z.-Y. Wu , J.-L. Zhang , S. Gao , W. Xiao , S. Du , K.-H. Ernst , H.-J. Gao , Angew. Chem. Int. Ed. 2020, 59, 17413–17416. 10.1002/anie.202005425 32603012

[14] C. Wäckerlin , J. Li , A. Mairena , K. Martin , N. Avarvari , K.-H. Ernst , Chem. Commun. 2016, 52, 12694–12697. 10.1039/c6cc05849c 27722304

[15] J. Li , K. Martin , N. Avarvari , C. Wäckerlin , K.-H. Ernst , Chem. Commun. 2018, 54, 7948–7951. 10.1039/c8cc04235g 29955753

[16] A. Mairena , M. Baljozović , M. Kawecki , K. Grenader , M. Wienke , K. Martin , L. Bernard , N. Avarvari , A. Terfort , K.-H. Ernst , C. Wäckerlin , Chem. Sci. 2019, 10, 2998–3004. 30996879 10.1039/c8sc04720k PMC6430192

[17a] O. Stetsovych , M. Švec , J. Vacek , J. V. Chocholoušová , A. Jancarik , J. Rybáček , K. Kosmider , I. G. Stará , P. Jelínek , I. Starý , Nat. Chem. 2017, 9, 213–218; 28221353 10.1038/nchem.2662

[17b] K.-H. Ernst , Nat. Chem. 2017, 9, 195–196. 28221349 10.1038/nchem.2728

[18] J. Barth , H. Brune , G. Ertl , R. Behm , Phys. Rev. B 1990, 42, 9307–9318. 10.1103/physrevb.42.9307 9995168

[19] J. Björk , S. Stafström , F. Hanke , J. Am. Chem. Soc. 2011, 133, 14884–14887. 21859135 10.1021/ja205857a

[20] P. V. C. Medeiros , G. K. Gueorguiev , S. Stafström , Phys. Rev. B 2012, 85, 205423/1–7.

[21] D. G. de Oteyza , A. Garcia-Lekue , M. Vilas-Varela , N. Merino-Diez , E. Carbonell-Sanroma , M. Corso , G. Vasseur , C. Rogero , E. Guitian , J. I. Pascual , J. E. Ortega , Y. Wakayama , D. Pena , ACS Nano 2016, 10, 9000–9008. 27548516 10.1021/acsnano.6b05269 PMC5043421

[22] N. Merino-Díez , M. S. G. Mohammed , J. Castro-Esteban , L. Colazzo , A. Berdonces-Layunta , J. Lawrence , J. I. Pascual , D. G. de Oteyza , D. Peña , Chem. Sci. 2020, 11, 5441–5446. 34094071 10.1039/d0sc01653e PMC8159356

[23] A. Keerthi , C. Sánchez-Sánchez , O. Deniz , P. Ruffieux , D. Schollmeyer , X. Feng , A. Narita , R. Fasel , K. Müllen , Chem. Asian J. 2020, 15, 3807–3811. 32955160 10.1002/asia.202001008 PMC7756733

[24] S. Blankenburg , J. P. Ruffieux , R. Jaafar , D. Passerone , X. Feng , K. Müllen , R. Fasel , C. A. Pignedoli , ACS Nano 2012, 6, 2020–2025. 22324827 10.1021/nn203129a

[25] A. Mairena , C. Wäckerlin , M. Wienke , K. Grenader , A. Terfort , K.-H. Ernst J. Am. Chem. Soc. 2018, 140, 15186–15189. 30383363 10.1021/jacs.8b10059

