# Supplementary material for: Stereospecific on‐Surface Cyclodehydrogenation of Bishelicenes: Preservation of Handedness from Helical to Planar Chirality
Source: Chemistry. 2021 Aug 26;27(54):13523–6. doi: 10.1002/chem.202102069 (PMC8518606; doi:10.1002/chem.202102069)
Supplement: Supplementary file 1 — Supporting Information [file CHEM-27-13523-s001.pdf]

Chemistry–A European Journal

Supporting Information

Stereospecific on-Surface Cyclodehydrogenation of Bishelicenes: Preservation of Handedness from Helical to Planar Chirality

Bahaaeddin Irziqat[+], Aleksandra Cebrat[+], Miloš Baljozović, Kévin Martin, Manfred Parschau, Narcis Avarvari, and Karl-Heinz Ernst*

Table of Contents

Experimental Procedures...2
Results and Discussions...5

Experimental Procedures

Chemical synthesis of 2,2’-bispentahelicene (1)

General Information:

All reagents and chemicals from commercial sources were used without further purification. Solvents were dried and purified using standard techniques. Column chromatography was performed with analytical-grade solvents using Aldrich silica gel (technical grade, pore size 60 Å, 230-400 mesh particle size). Flexible plates ALUGRAM® Xtra SIL G UV254 from MACHEREY-NAGEL were used for TLC. Compounds were detected by UV irradiation (Bioblock Scientific) or staining with iodine, unless otherwise stated.

NMR spectra were recorded with a Bruker AVANCE III 300 (^{1}H, 300 MHz and ^{13}C, 76 MHz) and Bruker AVANCE DRX 500 (^{1}H, 500 MHz and ^{13}C, 125 MHz). Chemical shifts are given in ppm relative to tetramethylsilane TMS and coupling constants *J* in Hz. Residual non-deuterated solvent was used as an internal standard.

Synthetic Procedures:

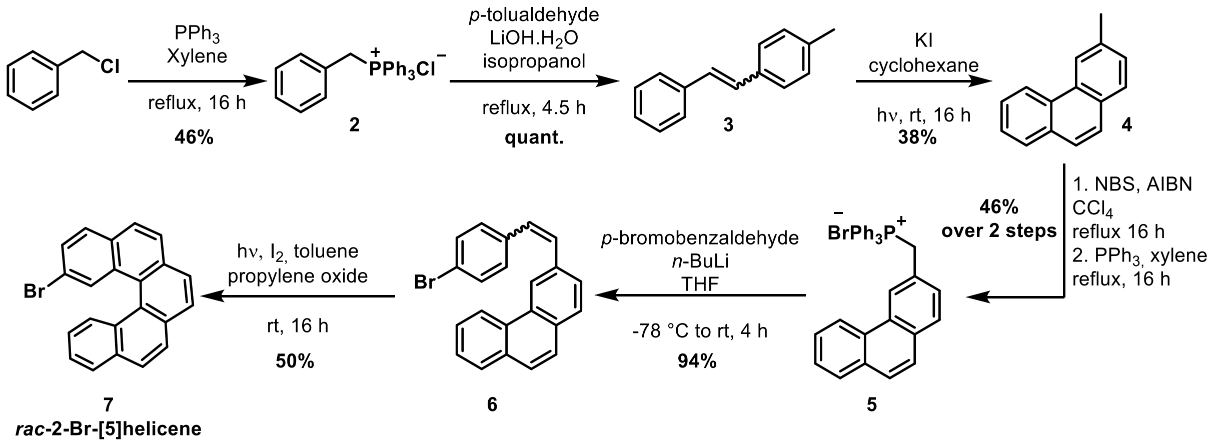

rac-2-Br-[5]helicene (7)

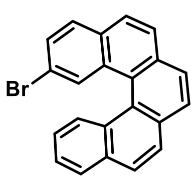

Compound 7 has been synthesized in six steps from chloromethyl-benzene according to the published method with 4% overall yield.[1,2]

Stilbene 6 (0.6 g, 1.67 mmol, 1 equiv) and iodine (0.45 g, 1.75 mmol, 1.05 equiv) were dissolved in toluene (650 mL). The solution was degassed for 15 min, and then propylene oxide (5.84 mL, 83.5 mmol, 50 equiv) was added. The mixture thus obtained was irradiated under stirring for 16 h with a Hg lamp (150 W). The synthesis was replicated in two batches, for a total amount of 1.2 g of stilbene compound. After evaporation of toluene, the crude was purified by chromatography over silica gel column (petroleum ether/DCM, 9/1, Rf = 0.46). 0.6 g (50% yield) of 2-Br-[5]helicene (7) were obtained as a light yellow powder.

^{1}H NMR (300 MHz, Chloroform-*d*) δ 8.69 (d, *J* = 1.6 Hz, 1H), 8.52 (d, *J* = 8.5 Hz, 1H), 8.01 – 7.90 (m, 2H), 7.92 – 7.83 (m, 5H), 7.82 (d, *J* = 8.6 Hz, 1H), 7.64 – 7.50 (m, 2H), 7.35 (ddd, *J* = 8.4, 6.9, 1.4 Hz, 1H).

MALDI-TOF = 356.2

The spectral data for this compound match those reported in the literature.[2]

rac-2,2'-bis-[5]helicene (1)

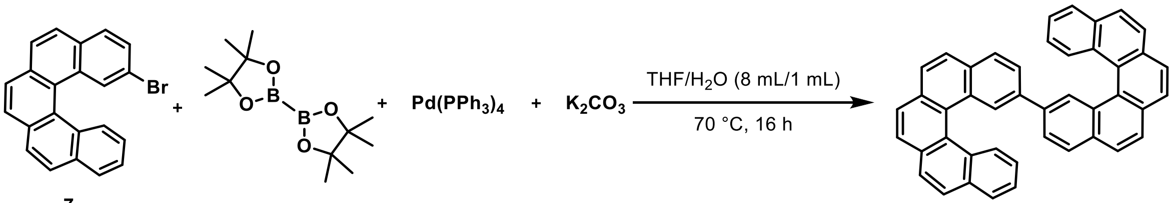

7

1

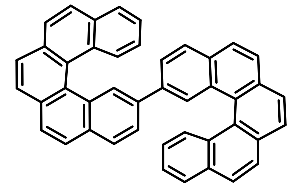

A synthetic strategy[3] involving the homo-coupling of aromatic bromides in the presence of bis(pinacolato)diboron has been adapted for the preparation of bis(pentahelicene) **1**. In a Schlenk flask 2-Br-[5]helicene (**7**) (100 mg, 0.28 mmol, 1 equiv), bis(pinacolato)diboron (35.5 mg, 0.14 mmol, 0.5 equiv) and $Pd(PPh_3)_4$ (9.7 mg, 8.4 µmol, 0.03 equiv) were dissolved in THF (8 mL). The solution was then bubbled with argon for 15 min, followed by the addition of a solution of potassium carbonate (116.1 mg, 0.84 mmol, 3 equiv) in water (1 mL). The mixture was stirred at 70 °C for 16 hours and then allowed to reach the room temperature, followed by the addition of 20 mL of CH_2Cl_2. The organic layer was washed three times with water, three times with brine and then dried over $MgSO_4$. The crude product was concentrated under vacuum and then purified by column chromatography on silica gel (PE/DCM as eluent 8/2 and then 6/4 gradient) to afford 15 mg (10% yield) of 2,2'-bis[5]helicene (**1**) as a white solid.

^{1}H NMR (500 MHz, 55 °C, Chloroform-*d*) δ δ 8.70 (d, *J* = 1.6 Hz, 2H), 8.67 (d, *J* = 8.5 Hz, 2H), 8.65 (d, *J* = 1.6 Hz, 2H), 8.49 (d, *J* = 8.5 Hz, 2H), 7.97 – 7.95 (m, 2H), 7.95 – 7.93 (m, 3H), 7.90 (d, *J* = 8.6 Hz, 3H), 7.89 – 7.88 (m, 2H), 7.88 – 7.86 (m, 10H), 7.86 – 7.84 (m, 9H), 7.83 – 7.80 (m, 3H), 7.63 (dd, *J* = 8.4, 1.8 Hz, 2H), 7.52 (dd, *J* = 8.3, 1.7 Hz, 2H), 7.47 (ddd, *J* = 8.0, 6.8, 1.1 Hz, 2H), 7.33 (ddd, *J* = 8.4, 6.8, 1.4 Hz, 2H), 7.24 (d, *J* = 1.1 Hz, 2H), 6.85 (ddd, *J* = 8.3, 6.8, 1.4 Hz, 2H).

^{13}C NMR (126 MHz, 55 °C, Chloroform-*d*) δ 137.97, 136.51, 132.99, 132.91, 132.83, 132.77, 132.58, 132.56, 132.13, 132.01, 131.85, 131.30, 131.28, 131.21, 131.15, 131.11, 130.99, 128.89, 128.72, 128.55, 128.44, 128.39, 128.00, 127.93, 127.85, 127.73, 127.56, 127.54, 127.51, 127.38, 127.31, 127.30, 127.19, 126.55, 126.51, 126.50, 126.47, 126.44, 126.15, 125.65, 124.78, 124.75.

HRMS: 554.2030

^{1}H NMR 2,2'-bis(pentahelicene) 1
The colored bands tentatively assign the different diastereomers (*M,P*)- + (*P,M*)-bis[5]helicene and (*M,M*)- + (*P,P*)-bis[5]helicene, which are present at a ratio of 1:1.

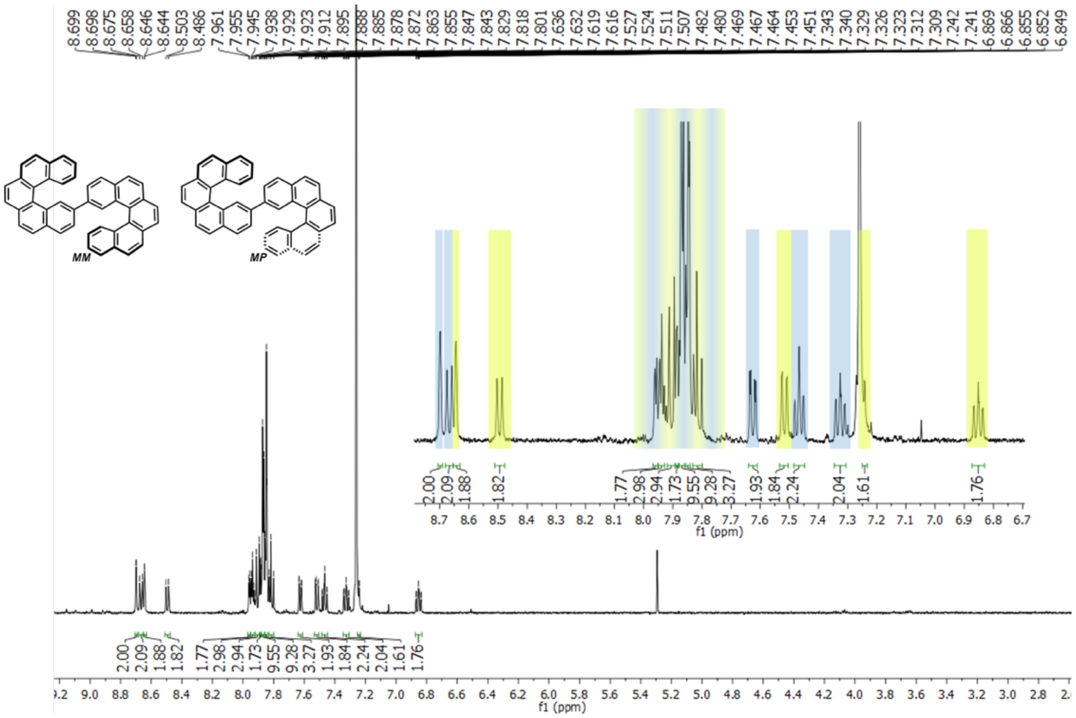

^{13}C NMR 2,2'-bis(pentahelicene) 1

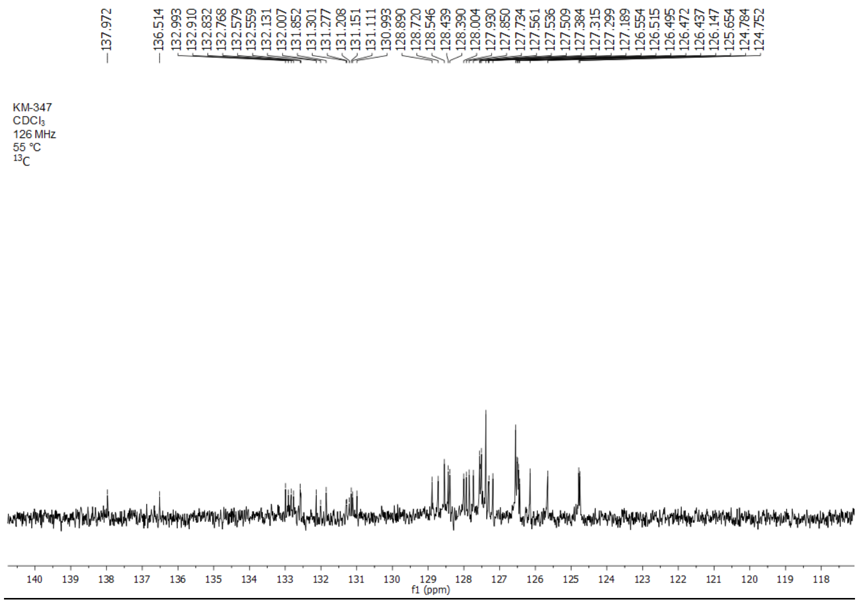

Experimental details:

Sample Preparation: The Au(111) single crystal was *in vacuo* prepared by repeated cycles of standard argon-ion bombardment and subsequent annealing at 600 °C for 15 min until a clean surface was obtained, as judged by STM. Precursor racemic bis[5]H molecules were thermally deposited from a Knudsen-cell type evaporator held at 240 °C onto a cleaned Au(111) surface , which was kept at 100 °C throughout the deposition. Different coverages were adjusted by different exposure times.

Characterization: STM experiments were carried out under ultrahigh vacuum conditions (base pressure < 10^{-10} mbar) using variable-temperature STM (Omicron Nanotechnology GmbH) operated at 50 K and a home-built low-temperature STM operated at 7 K. The 7 K studies were performed in order to confirm homochiral assembly of the cyclodehydrogenated product. All STM measurements were acquired in constant-current mode using electrochemically etched tungsten tips with the bias voltage applied to the sample. Tip shaping was performed by poking it slightly into the bare Au surface so that its apex was coated with Au atoms (i.e., Au-terminated tip). The thermally induced cyclodehydrogenation of the racemic bis[5]H into a planar coronocoronene was achieved by annealing the sample to ~400 °C for 30 min and subsequently cooled to cryogenic temperatures for analysis. The submolecular resolution of the planar coronocoronene molecules was most likely achieved by unintentional pick up of a single CO molecule from the surface. Measured images were analyzed using the WSxM software and filtered by global plane subtraction.
In addition: Fig. 2d & Fig. 3 row-wise offset flatten filter + 2D FFT filter to reduce the noise.
All used filters have no effect on dimensions, shape, and brightness difference of the resolved features.
The polarity stated in the parameters means that positive bias voltages indicate tunneling from the STM tips into the unoccupied surface states.

ToF-SIMS (IONTOF, ToF-SIMS 5) spectra were recorded with a 25 keV beam of Bi_3^+ primary ions. The beam was randomly rasterized over an area of 0.5 × 0.5 mm^2. An extraction voltage of 3 kV was used. The mass calibration was performed using the Au_n^+ peaks of the substrate. All measurements were performed in ultrahigh vacuum (p < 5 × 10^{-9} mbar).

Computational details:
The optimized structures and the relative molecular alignments of single adsorbates and dimers of bis[5]H and coronocoronene were computed with the molecular mechanics AMBER-type force field of HyperChem 8.0 program. A four-layer Au(111) slab with periodic boundary conditions was used as template, with the gold atoms fixed in space during the calculations. The molecules were enabled to move freely during the optimization. For the single molecule and the dimers 108 and 216 different initial configurations (x, y, z coordinates, z = axis normal to the gold surface) were tested, respectively. For the geometry optimization, a conjugate Polak-Ribiere gradient was used with a termination gradient smaller than 10^{-4} kcal·Å^{-1}·mol^{-1}. Dimer binding energies were calculated by taking the surface binding energy of a single molecule times two minus the dimer binding energy on the surface. Molecular frontier orbitals (HOMO −9 to HOMO) were simulated directly on the force field-optimized structures using semiempirical extended Hückel theory.

Results and Discussion

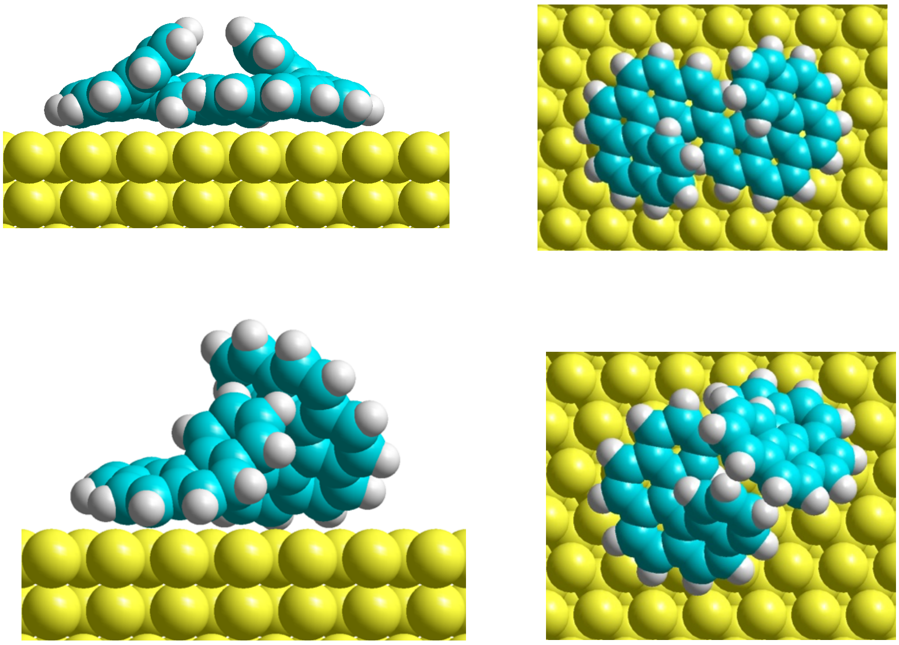

Figure S1. Top and side view of single AMBER force field-relaxed *(P,P)*-bis[5]H (top) and *(M,P)*- bis[5]H (bottom) on Au(111). The *meso-(M,P)*-bis[5]H lies 6.5 kcal/mol higher in energy than the other diastereomers.

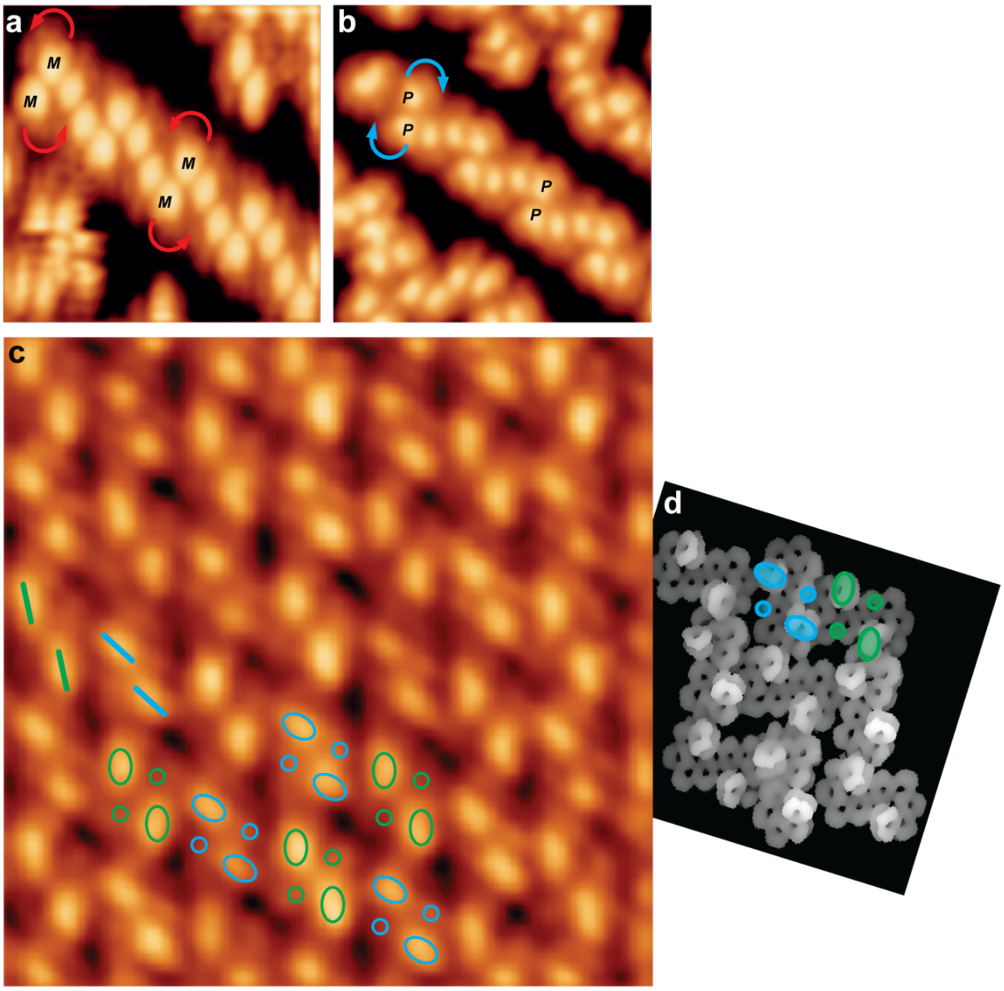

Figure S2. Analysis of handedness in the STM pattern at intermediate and full monolayer coverage of bis[5]H. (a) STM image (10 nm × 10 nm U = –2.5 V, I = 20 pA, T = 50 K of a zigzag row with a (*M,M*)-[5]H molecule at the end. (b) STM image (10 nm × 10 nm U = –2.3 V, I = 20 pA, T = 50 K of a zigzag row with a (*P,P*)-[5]H molecule at the end. (c) STM image (10 nm × 10 nm U = –2.5 V, I = 20 pA, T = 50 K) showing different contrast for every second molecule in a row. Bars, circles and ellipses highlight the dominant features and are related by a reflection plus 60° rotation operation. (d) STM contrast of unoccupied states based on the model shown in Figure S3. The four-lobe contrast appearance is again highlighted in order to show the agreement with the experiment. Hence, it is concluded that a 2D racemate is present.

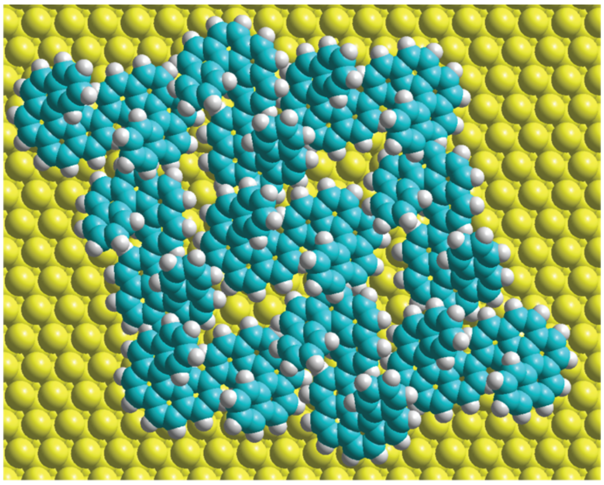

Figure S3. Tentative model for the 2D racemate crystal at monolayer coverage accounting for enantiomer alternation plus 60° rotation. Due to partial intermolecular overlap the van-der-Waals contact in this model is maximized.

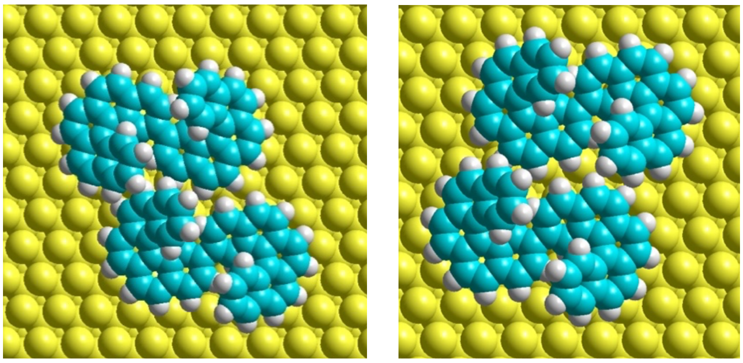

Figure S4. Top view of AMBER force field-relaxed van der Waals dimers of bis[5]H on Au(111). A heterochiral dimer (left) is favored by 0.6 kcal/mol.

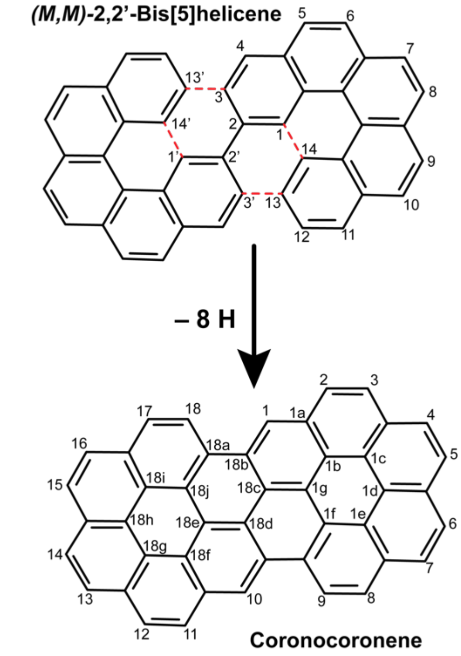

Figure S5. Sketch of intramolecular cyclodehydrogenation of bis[5]H to coronocoronene. Hydrogen atoms are released from — and C-C coupling occurs at — C-atoms 3-13', 1'-14', 1-14, and 3'-13, as indicated by red dashed lines.

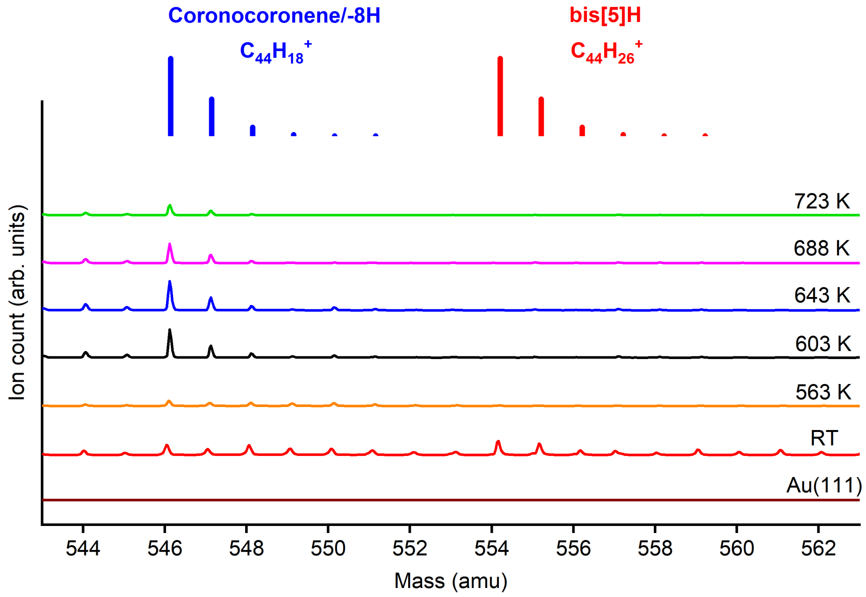

Figure S6. ToF-SIM spectra of a saturated monolayer of bis[5]H on Au(111). The mass distributions of bis[5]H ($C_{44}H_{26}^{+}$) and coronocoronene (bis[5]H/–8H, $C_{44}H_{18}^{+}$) are shown as colored bars. The dehydrogenation reaction occurs at 563 K. Further annealing to 603 K, leads to complete dehydrogenation and planarization, resulting in the loss of 8 hydrogen atoms per molecule and formation of coronocoronene.

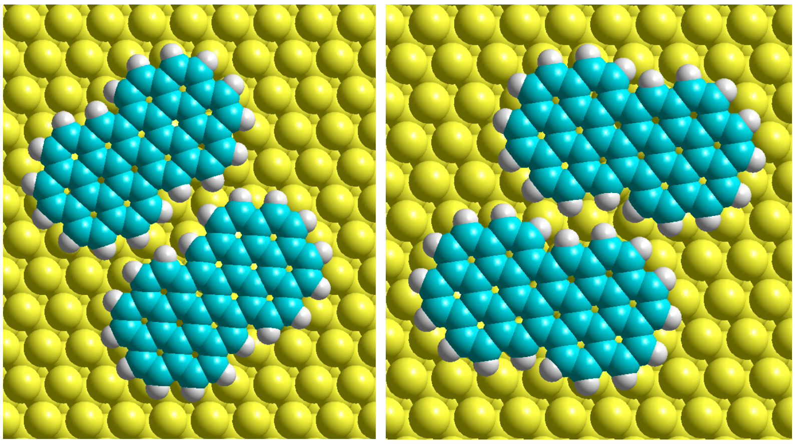

Figure S7. Comparison of force field-optimized heterochiral (left) and homochiral (right) dimers of coronocoronene. Formation of a homochiral dimer is favored by 0.72 kcal/mol.

Table S1. Force-field analysis of reaction educt, intermediate and final product. Comparison of the adsorption energy of bis[5]H, semi-planarized (bis[5]–4H) and coronocoronene **2** (bis[5]–8H), as result of the difference of the relaxed free molecule and the relaxed surface bound species.

	(*MM*)-bis[5]H free	(*MM*)-bis[5]H adsorbed	(*MM*)-bis[5]H / –4H free	(*MM*)-bis[5]H / –4H adsorbed	(*MM*)-bis[5]H / –8H free	(*MM*)-bis[5]H / –8H adsorbed
Energy [kcal/mol]	32.88	12.13	28.27	3.38	21.84	–5.13
ΔE ± 1 [kcal/mol]		–20.75		–24.89		–27.0

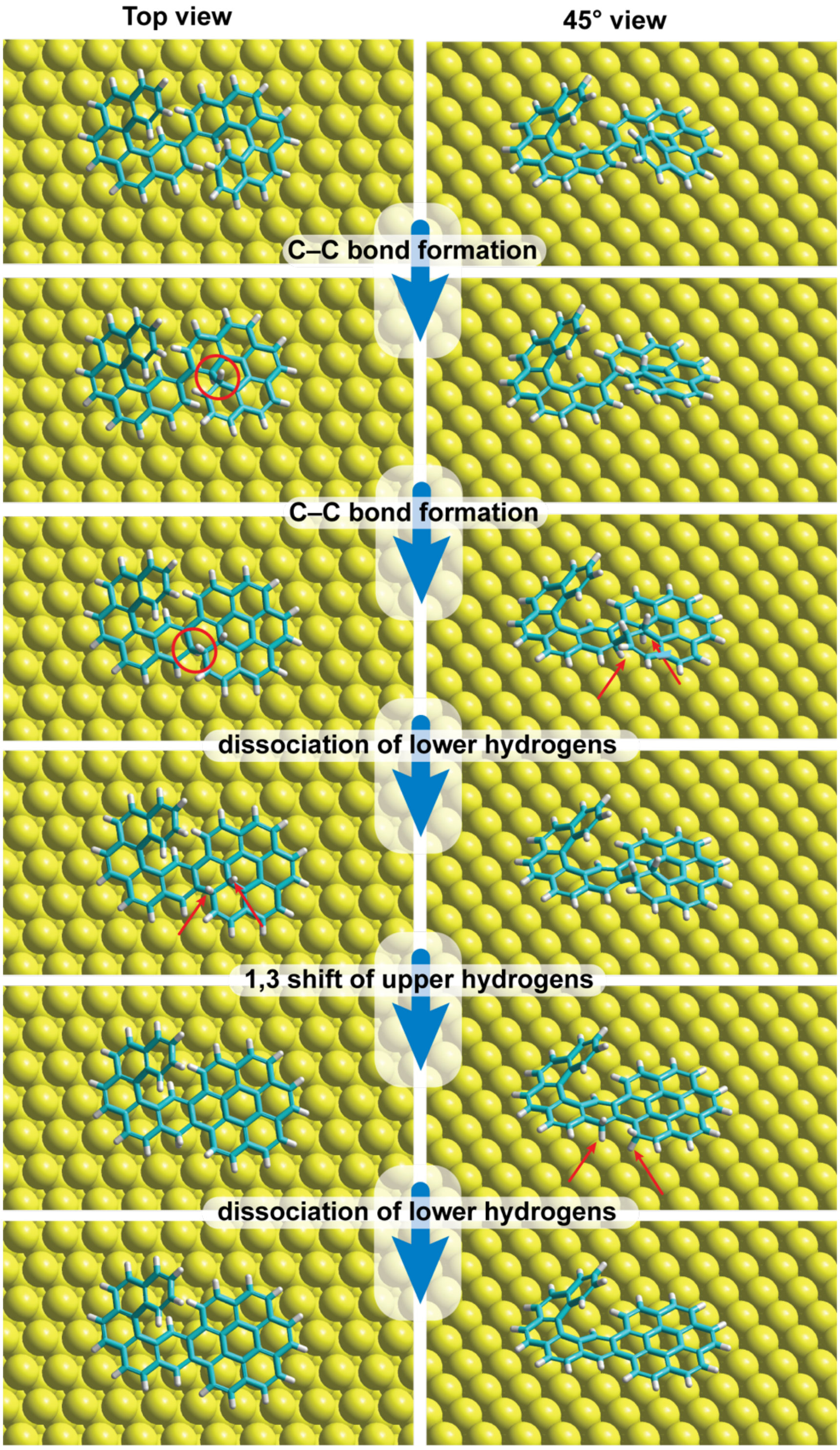

Figure S8. Sketch of the stepwise cyclodehydrogenation mechanism. Followed by C–C coupling between distal and proximal parts of one [5]H unit (red ellipses), dissociation of low-lying H-atoms from the molecule occurs. The upper H-atoms undergo a 1,3 shift such that CH_2 groups form (red arrows). The low-lying H-atoms of these CH_2 groups dissociate away from the molecule.

References

[1] T. Matsushima, S. Kobayashi, S. Watanabe, *J. Org. Chem.* **2016**, *81*, 7799–7806.
[2] R. El Abed, B. Ben Hassine, J.-P. Genêt, M. Gorsane, A. Marinetti, *Eur. J. Org. Chem.* **2004**, 1517–1522.
[3] K. H.Hendriks, W. Li, G. H. L. Heintges, G. W. P. van Pruissen, M. M. Wienk, R. A. J. Janssen, *J. Am. Chem. Soc.* **2014**, *136*, 11128–11133.
